# Cofactor Requirement of HpyAV Restriction Endonuclease

**DOI:** 10.1371/journal.pone.0009071

**Published:** 2010-02-05

**Authors:** Siu-Hong Chan, Lars Opitz, Lauren Higgins, Diana O'loane, Shuang-yong Xu

**Affiliations:** Research Department, New England Biolabs, Inc., Ipswich, Massachusetts, United States of America; Massachusetts Institute of Technology, United States of America

## Abstract

**Background:**

*Helicobacter pylori* is the etiologic agent of common gastritis and a risk factor for gastric cancer. It is also one of the richest sources of Type II restriction-modification (R-M) systems in microorganisms.

**Principal Findings:**

We have cloned, expressed and purified a new restriction endonuclease HpyAV from *H. pylori* strain 26695. We determined the HpyAV DNA recognition sequence and cleavage site as CCTTC 6/5. In addition, we found that HpyAV has a unique metal ion requirement: its cleavage activity is higher with transition metal ions than in Mg^++^. The special metal ion requirement of HpyAV can be attributed to the presence of a HNH catalytic site similar to ColE9 nuclease instead of the canonical PD-X-D/EXK catalytic site found in many other REases. Site-directed mutagenesis was carried out to verify the catalytic residues of HpyAV. Mutation of the conserved metal-binding Asn311 and His320 to alanine eliminated cleavage activity. HpyAV variant H295A displayed approximately 1% of wt activity.

**Conclusions/Significance:**

Some HNH-type endonucleases have unique metal ion cofactor requirement for optimal activities. Homology modeling and site-directed mutagenesis confirmed that HpyAV is a member of the HNH nuclease family. The identification of catalytic residues in HpyAV paved the way for further engineering of the metal binding site. A survey of sequenced microbial genomes uncovered 10 putative R-M systems that show high sequence similarity to the HpyAV system, suggesting lateral transfer of a prototypic HpyAV-like R-M system among these microorganisms.

## Introduction

Restriction-modification (R-M) systems that recognize and cleave DNA in a highly specific manner are ubiquitous in prokaryotic microorganisms (and their viruses) [1]. *Helicobacter pylori*, the etiologic agent of common gastritis and a risk factor for gastric cancer [2], curiously is one of the richest sources of Type II restriction-modification (R-M) systems in any living organisms [3], [4]. The extraordinary number of Type II R-M systems appears to be the result of *H. pylori'*s natural competency of transformation by exogenous DNA [4]–[6]. In addition to defense against invading phages, evidence has suggested that the MTases (within active R-M system [7] or orphan MTases [8], [9]) are involved in transcriptional regulation of other genes akin to the epigenetics of mammalian cells.

Genome mining of sequenced microbial genomes has resulted in a wealth of restriction enzymes with new specificities or unique properties (ApeKI (G∧CWGC), PhoI (GG∧CC), CviKI-1 (RG∧CY), NmeAIII (GCCGAG 20–21/18–19) [10], [11], Nt.CviPII (∧CCD) [12]; NEB catalog 2009/10) [1]. The goal of this work was to clone, express, purify and characterize HpyAV restriction endonuclease (REase), which is one of the putative R-M systems from *H. pylori* 26695 [3]. During the purification process, we found that Ni^++^ has a stimulatory effect on HpyAV activity. Bioinformatics analysis showed that HpyAV contains a HNH catalytic site highly similar to that of colicin E9 (ColE9). Sequence alignment of HpyAV and ColE9 and other HNH nucleases identified four highly conserved catalytic residues. By site-directed mutagenesis we confirmed that these residues are important for DNA cleavage. In addition to Ni^++^, we found that HpyAV is also active in Mn^++^ and Co^++^. We therefore surveyed a few other HNH REases and found that KpnI is also active in a multitude of transition metals. Finally, a BLASTP search in sequenced bacterial genomes revealed ten putative HpyAV R-M systems. These microorganisms reside within human bodies or in mammals that are closely associated with humans, suggesting a possible lateral transfer mechanism.

## Results

### The HpyAV R-M System

**Figure 1:**
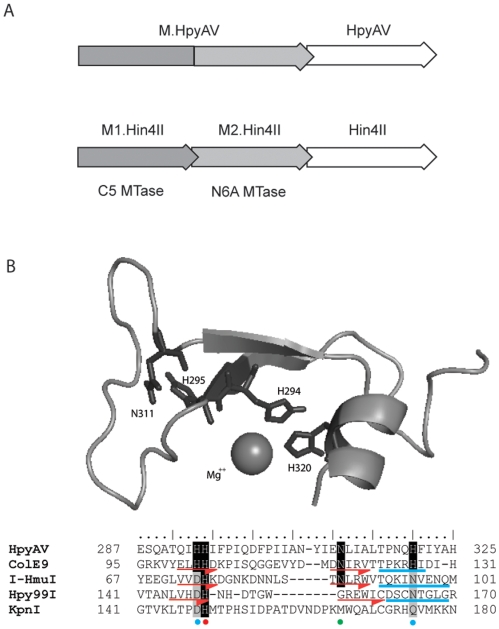
Gene organization of the HpyAV/Hin4II R-M systems and the structural model of the HpyAV catalytic site. **A.** Organization of the HpyAV and Hin4II R-M systems. The Hin4II R-M system consists of separate M1 and M2 genes for C5 cytosine (dark grey) and N6 adenine (light grey) methylation, respectively, preceding the ORF of Hin4II [80]. M.HpyAV is a fusion of C5 and N6A MTase domains with high sequence similarity to M1.Hin4II and M2.Hin4II, respectively. **B.** The structural model of HpyAV catalytic site and structural alignment to HNH endonucleases. Modeling of amino acid residues 281 to 360 of HpyAV to the ββα-Me motif of ColE9 and the structural alignment to ColE9, I-HmuI, Hpy99I and KpnI were done as described in Materials and Methods. The blue dots underneath the alignment indicate metal-binding residues; the red dots indicate the general base His and the green dot indicates the Asn implied to decrease the pKa of the general base His in ColE9 and I-HmuI. Amino acid residues that are assigned to β-strands and the helix of the ββα-Me motif are indicated by red arrows and blue rods, respectively. The conserved residues implicated in catalysis are colored in black or grey.

Restriction mapping and run-off sequencing results indicated that the native HpyAV REase isolated from *Helicobacter pylori* strain 26695 recognizes the asymmetric target sequence CCTTC and cleaves 6 nt and 5 nt downstream of the top strand and the bottom strand, respectively (CCTTC 6/5; data not shown). Enzymes that recognize asymmetric sequences frequently require two methyltransferases (MTases) to modify the two strands of DNA. In the case of HpyAV the MTase(s) must modify a C of the top strand and an A of the bottom strand in the target sequence. From the genomic sequence of *H. pylori* 26695 (Genbank nucleotide accession NC_000915), the R gene (hp_0053) of the HpyAV R-M system is located downstream of the M gene (hp_0054) and runs in the same direction as the M gene (**Fig. 1A**). In addition, the M gene of the HpyAV R-M system is a fusion of a C5 cytosine MTase and a N6 adenine MTase highly homologous to M1.Hin4II and M2.Hin4II, respectively (**Fig. 1A**). We re-sequenced the junction of the two MTase domains from the cloned M gene and from a PCR product derived from the genomic DNA and found no stop codon between the two domains, confirming that M.HpyAV is a true fusion of C5 cytosine MTase and N6 adenine MTase, although the size of the translation product has not been confirmed biochemically. An over-expression *E. coli* strain was constructed by transforming *E. coli* ER3081 (NEB) with pSYX20-*hpyAVM* and pAII17-*hpyAVR* by sequential transformation (See Materials and Methods).

### Metal Ion Requirement for DNA Cleavage

**Figure 2:**
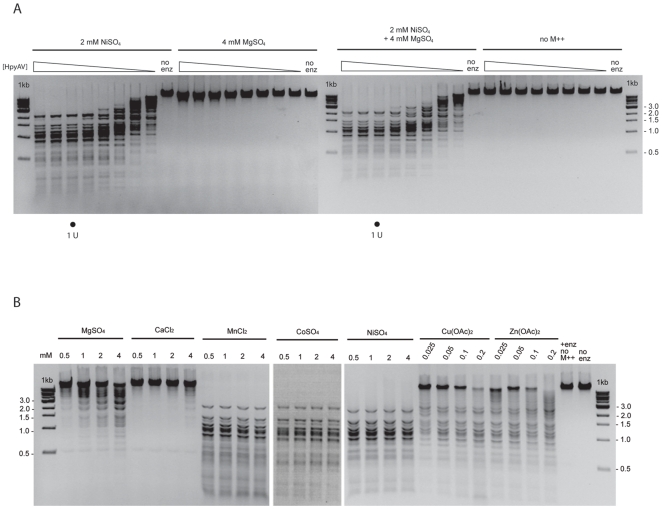
HpyAV endonuclease activity in buffers with various divalent cations. **A.** Cleavage activity of HpyAV in MgSO_4_ and NiSO_4_. Decreasing concentrations of HpyAV were added to reactions containing 1 µg of λ DNA, 20 mM Tris-HCl, pH 7.9, 200 mM NaCl supplemented with 2 mM of NiSO_4_, 4 mM of MgSO_4_, 2 mM of NiSO_4_ and 4 mM of MgSO_4_ or no divalent metal ions. The reactions were carried out as described in Materials and Methods. The reactions that exhibit 1 U of HpyAV activity (complete cleavage) are marked with a dot. **B.** DNA cleavage activity of HpyAV in buffers supplemented with the indicated concentration of metal ion solutions. Eight units of HpyAV were incubated with 1 µg of λ DNA in 20 mM Tris-HCl, 200 mM NaCl as described in Materials and Methods.

The purified recombinant HpyAV exhibited very low cleavage activity on λ DNA in the standard reaction condition containing 4 mM MgSO_4_ (**Fig. 2A**). However, when 2 mM NiSO_4_ was added into the cleavage reaction in the presence or absence of 4 mM MgSO_4_, HpyAV exhibited equally high cleavage activity (**Fig. 2A**). This shows that HpyAV prefers Ni^++^ for cleavage activity. This discovery prompted us to examine HpyAV against other divalent metal ions including Ca^++^, an alkaline earth metal ion that is inhibitory to REases containing the canonical PD-X-(D/E)XK catalytic motif, and ions of other transition metals in the same period (Mn^++^, Co^++^, Cu^++^ and Zn^++^). We found that HpyAV showed a different degree of cleavage activity with divalent metal ions. It was most active with MnCl_2_, NiSO_4_ or CoSO_4_ - complete cleavage of λ DNA was achieved with 0.5 to 4 mM of these three metal ions (**Fig. 2B**). For Cu(OAc)_2_ and Zn(OAc)_2_, concentrations higher than 2 mM were inhibitory to HpyAV endonuclease activity (data not shown), and complete cleavage was not obtained under the assay conditions. HpyAV showed much lower activity in the presence of CaCl_2_ or MgSO_4_ (**Fig. 2B**). Table 1 summarizes the specific activity of HpyAV with various metal ions. HpyAV is equally active in MnCl_2_, CoSO_4_ and NiSO_4_ (specific activities are within a 2-fold margin for a 2-fold dilution series of the enzyme), and complete cleavage of λ DNA was not achieved in the presence of MgSO_4_, CaCl_2_, Cu(OAc)_2_ or Zn(OAc)_2_ at the highest enzyme concentration available (40 µmol of HpyAV on 0.3 pmol (1 µg) of λ DNA). By comparing the cleavage patterns, HpyAV is estimated to exhibit less that 0.4% of cleavage activity in buffers with MgSO_4_ and CaCl_2_, less than 6% with Cu(OAc)_2_ and less than 0.8% with Zn(OAc)_2_.

**Table 1:** Specific activity of HpyAV and KpnI.

	HpyAV		KpnI	
	Sp. Activity (U/mg) a	%	Sp. Activity (U/mg) a	%
Mg^++^	<<200	<0.4^b^	282000	100
Ca^++^	<<200	<0.4^b^	7000	2.5
Mn^++^	2000	200	N/D^c^	N/D^c^
Co^++^	830	83	42000	15
Ni^++^	1000	100	32000	10
Cu^++^	<200	<6^b^	N/D^c^	N/D^d^
Zn^++^	<200	<0.8^b^	28000	10

a Specific activity are average values of triplicate experiments for HpyAV and duplicate experiments for KpnI.

b Complete cleavage of substrate DNA was not achieved with the highest available concentration of HpyAV. Percentage activity was estimated by comparing the cleavage pattern of the highest concentration of HpyAV with the indicated metal ion to the matching pattern with Ni^++^ (data not shown).

b The specific activity of KpnI was not determined in Mn^++^ because star activity appeared before complete cleavage of the substrate DNA was achieved.

d Specific activity of KpnI was not determined in Cu^++^ because the same incomplete cleavage pattern was observed over a 120-fold difference in enzyme concentration.

### Homology Modeling of Catalytic Residues

The strong stimulation effect of Ni^++^ on endonuclease activity is unique to HpyAV. It led us to examine the HpyAV amino acid sequence in more details. HpyAV is not homologous to any known REases except its isoschizomer Hin4II (data not shown). Manual examination of the amino acid sequence of HpyAV revealed a HNH catalytic motif highly homologous to that of colicin E9. Homology modeling of amino acid (aa) residues 287–325 of HpyAV to the ββα-Me motif of ColE9 (aa 95–131) resulted in a model free of clashes and with all the conserved catalytic residues (His102, His103, Asn118 and His127 in ColE9; H294, H295, N311, and H320 in HpyAV) structurally aligned to the HNH endonucleases including I-HmuI and Hpy99I (**Fig.** **1B**).

### Site-Directed Mutagenesis of the HNH Catalytic Site

**Figure 3:**
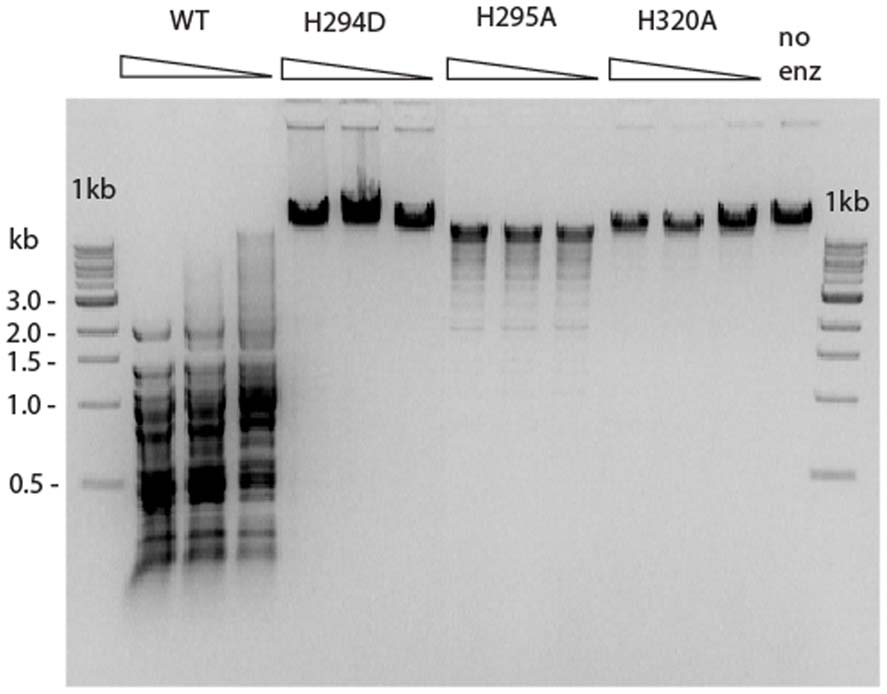
DNA cleavage activity of the catalytic residue mutants. Purified protein of WT, H294D, H295A and H320A were assayed as described in Materials and Methods in the presence of 2 mM NiSO_4_. Five μl of undiluted, three-fold and nine-fold dilutions of 1.5 mg/ml of enzyme solution were assayed on 1 µg of λ DNA.

From biochemical and structural studies of colicin E9, His103 acts as the general base to deprotonate a water molecule for the hydrolysis of the scissile phosphodiester bond. His102 and His127 coordinate the single divalent metal ion for transition state stabilization. Asn87 of I-HmuI and Asn118 of ColE9 are proposed to form a hydrogen bond to the general base His and increase its pKa for the activation of the nucleophilic water [13], [14]. In this study, the corresponding residues of HpyAV (His294, His295, Asn311 and His320) were mutated to verify their role in catalysis. Mutants H294D, H295A, and H320A were constructed and purified. H294D and H320A did not show any cleavage activity at up to 7.5 µg of protein (**Fig. 3** and data not shown) in the presence of 2 mM NiSO_4_ or MgSO_4_, indicating that (i) the removal of the imidazole group at position 320 eliminated cleavage activity; (ii) the negatively charged Asp (as found in I-HmuI, Hpy99I and KpnI at the same aa position; **Fig. 1B**) cannot replace the histidine residue at position 294 for metal coordination in HpyAV. It is somewhat unexpected that substitution of the general base His295 by Ala did not completely eliminate the cleavage activity (**Fig. 3**); H295A still retains approximately 1% of wt activity, suggesting that an alternative weaker general base exists in the catalytic site when the general base His295 is absent. To explore the consequence of other amino acid substitutions, we also mutated His295 to Lys, Asn or acidic resides Asp/Glu. IPTG-induced cell extracts expressing these four mutants (H295K, H295N, H295D, and H295E) did not show any cleavage activity (data not shown), indicating that Lys, Asn, Asp, or Glu residues cannot replace His295 in the catalytic site. Cell extract with N311A variant failed to show any detectable cleavage activity (data not shown). It is concluded that His294, H295, Asn311 and His320 are important residues for HpyAV endonuclease activity.

### KpnI Endonuclease Activity with Different Divalent Metal Ions

**Figure 4:**
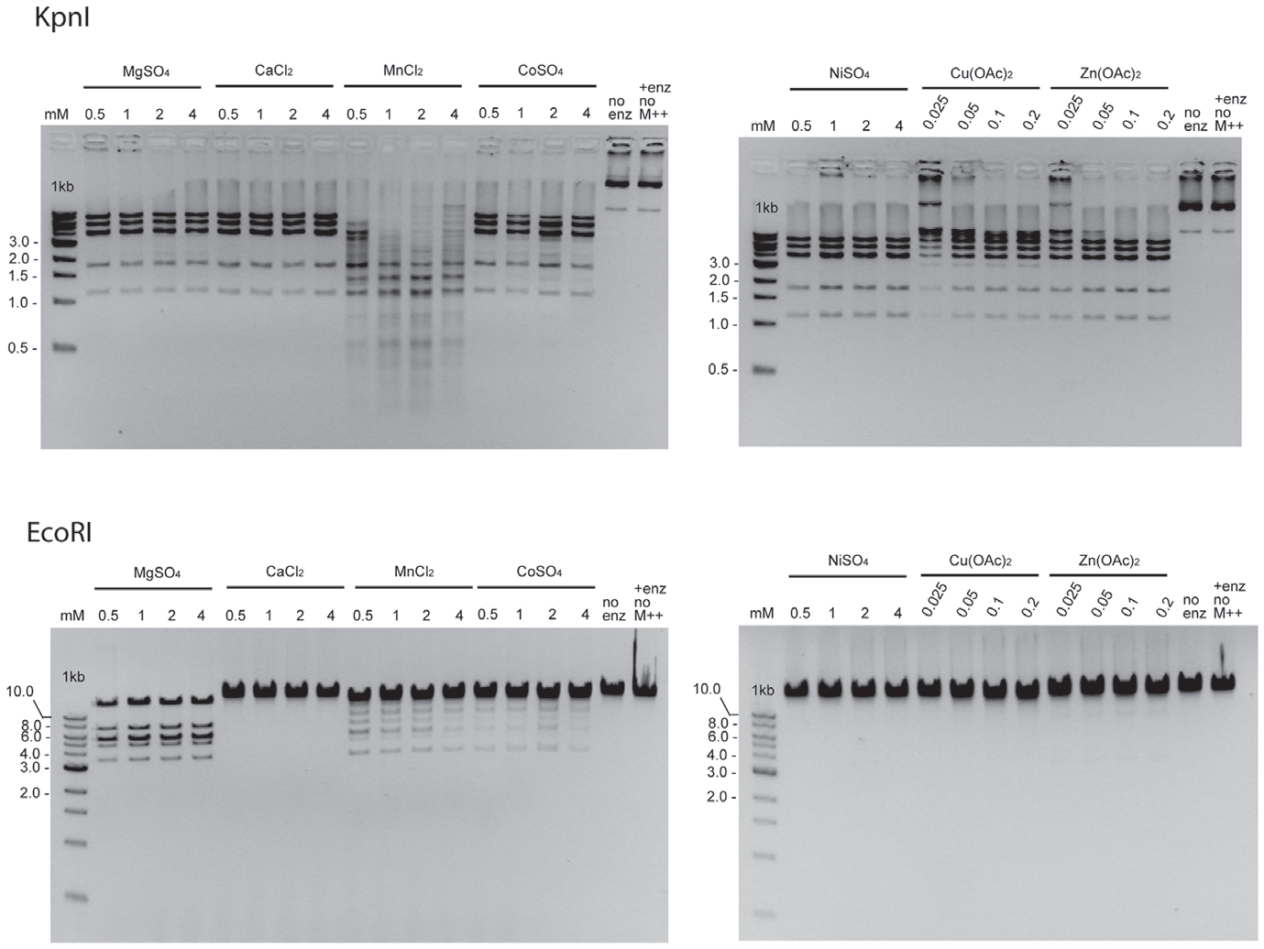
DNA cleavage activity of KpnI and EcoRI in the presence of different metal ions. Ten units of KpnI or EcoRI were incubated with 1 µg of pXba or λ DNA, respectively, as described in Materials and Methods.

KpnI is an HNH Type IIP REase that has been shown to be active with Mg^++^ or Ca^++^ [15], [16]. The high activity of HpyAV with transition metal ions prompted us to investigate if it is also true for KpnI. **Fig. 4** shows that KpnI is active in all of the transition metals tested. **Table 1** shows that the specific activity of KpnI is highest with MgSO_4_, followed by CoSO_4_ (15% of MgSO_4_), NiSO_4_ and Zn(OAc)_2_ (both 15% of MgSO_4_) and CaCl_2_ (2.5% of MgSO_4_). The specific activity could not be determined with MnCl_2_ because cleavage at non-cognate sites (star activity) was observed before complete cleavage of the cognate sites was achieved (data not shown). The specific activity in Cu(OAc)_2_ was also not determined because the same incomplete cleavage pattern was observed over a 120-fold difference in enzyme concentration (data not shown). As a control, 10 U of EcoRI, a canonical PD-(D/E)XK Type IIP REase, was also tested. Under the assay conditions, EcoRI was most active in MgSO_4_, with very low level of activity in MnCl_2_ and CoSO_4_ and no activity in CaCl_2_, NiSO_4_, Cu(OAc)_2_ or Zn(OAc)_2_. For all three enzymes, no cleavage activity was observed without the added divalent metal ions, indicating that all the activities observed were caused by the presence of the metal ion cofactors.

### Homologous HpyAV Systems from Sequenced Microbial Genomes

HpyAV is an isoschizomer of Hin4II. M.HpyAV contains a C5 cytosine and a N6 adenine MTase domain highly homologous to M1 and M2.Hin4II (**Fig. 1A**). Sequence analysis showed that the corresponding regions of M.HpyAV are 57% and 56% identical to M1 and M2.Hin4II, respectively (**Table 2**). The REases HpyAV and Hin4II also share 48% sequence identity (**Table 2**). A BLASTP search of the GenBank genomes discovered 10 putative R-M systems that are highly homologous to the HpyAV system. The MTases and REases of these homologous systems, along with those of HpyAV and Hin4II, are shown in **Table 2**. Except for the *Yersinia kristensenii* and *Vibrionales bacterium* SWAT-3 systems, the M gene precedes the R gene with both of them oriented in the same direction. Also, like the HpyAV system, all of these R-M systems contain single MTase that are fusions of C5 cytosine and N6 adenine MTases. Their high sequence similarity suggests that these putative R-M systems may share the same recognition sequence (CCTTC). It is also noticeable that these homologous R-M systems are mostly carried by infectious microorganisms of human or mammalian hosts closely associated with humans. However, there are two putative endonucleases (EsaSS23P  = 393 aa; EsaSS44P  = 385 aa) without companion MTases from shotgun-sequenced environmental samples that share significant amino acid sequence identity with HpyAV and Hin4II (EsaSS23P vs. HpyAV  = 34% aa sequence identity; EsaSS44P vs Hin4II  = 29% aa sequence identity). They may recognize CCTTC or similar target site with 1-bp difference.

**Table 2:** Homologous HpyAV R-M systems.

REase^a^	Length (aa)	% ID	MTase^1^	Length (aa)	% ID	Organism
HpyAV	419	100	M. HpyAV	823	100	*Helicobacter pylori* 26659
HpyPORF48P	423	92	M.HpyPORF48P	822	98	*Helicobacter pylori* P12
HpyGORF49P^b^	419	96	M.HpyGORF49P	823	97	*Helicobacter pylori* G27
CupORF1468P	339	50	M.CupORF1468P	817	64	*Campylobacter upsaliensis* RM3195
SeqZORF1536P	417	50	M. SeqZORF1536P	810	52	*Streptococcus equi* subsp. *zooepidemicus* MGCS10565
Hin4II	418	48	M1.Hin4II M2.Hin4II	387 445	57^c^ 56^c^	*Haemophilus influenzae* RFL4
BhyWAORF699P	386	43	M.BhyWAORF699P	812	43	*Brachyspira hyodysenteriae* WA1
Nme180ORF295P	296	31	M.Nme18ORF295P	862	42	*Neisseria meningitidis* FAM18
MmyLCORFBP	330	40	M. MmyLCORFBP	834	55	*Mycoplasma mycoides* subsp. *mycoides* LC str. GM12
YkrORF13790P	426	39	M.YkrORF13790P	826	44	*Yersinia kristensenii* ATCC 33638
Bst43183ORF2897P	390	33	M.Bst43183ORF2897P	856	43	*Bacteroides stercoris* ATCC 43183
VbaORF22060P	326	30	M.VbaORF22060P	820	43	*Vibrionales bacterium* SWAT-3

a Names as in REBASE. All REases, except HpyAV and Hin4II, are putative (names end with P).

b In the genomic sequence, HpyGORF49P contains a deletion that introduces a stop codon within the HNH catalytic site. The reported length is a theoretical number based on the re-introduction of the deleted nucleotide to the genomic sequence.

c Sequence identity for M1 and M2.Hin4II were calculated based on pair-wise alignment of segments of M.HpyAV that can be aligned to M1 and M2.Hin4II, respectively (Fig. 1).

## Discussion

### Metal Ion Cofactor Preference of HNH Endonucleases

It has been well-documented that for restriction endonucleases (REases) with the canonical PD-X-(D/E)XK catalytic site, Mg^++^ and Mn^++^ support catalysis but Ca^++^ only supports DNA binding. One to two coordinated metal ions have been observed in the catalytic site in the crystal structures of REases in the presence of substrate DNA. Catalytic mechanisms for one- and two- ion-induced cleavage have been proposed [17]–[20]. It has been generally accepted that most Type IIP REases use a two-metal ion mechanism where metal ion A deprotonates the nucleophilic water molecule and metal ion B stabilizes the pentavalent phosphoanion transition state and activates a water molecule for protonation of the leaving 3′-phosphate oxygen. Some REases (EcoRI and BglII), however, appear to use a one-metal ion mechanism where the metal ion occupies site 1 and coordinates a nucleophilic water molecule for the attack of the scissile phosphate, although it has been noted that the second metal ions might have escaped detection because the second metal ions tend to have low occupancy in other structures [21]–[23]. Recently, Pingoud and colleagues presented experimental evidence and MD simulation results to support a generalized one-metal ion catalytic mechanism where site 1 has a higher affinity to Mg^++^ and site 2 plays a modulating role in the cleavage activity [23].

Endonucleases that contain the ββα-Me motif, on the other hand, are only observed with one coordinated divalent metal ion in their catalytic sites. The ββα-Me motif adopts a V-shape conformation consisting of two beta-strands connected by a loop in one arm followed by a helix that constitutes the other arm. The ββα-Me motif is present in non-specific endonucleases such as *Serratia* nuclease [24], [25], *E. coli* defense nucleases colicin E7 [26], [27] and E9 [28], [29], Holliday junction resolving T4 endonuclease VII [30] and homing endonucleases I-PpoI of the His-Cys family [31], [32]. HNH enzymes are a sub-group of the ββα-Me family where the metal ion is coordinated by two negatively charged amino acid residues (two histidines in ColE9 [29], [33], [34]; a glutamate and a asparagine in I-HmuI [13], [14] and Hpy99I [35]) and the non-bridging oxygen of the scissile phosphodiester bond of the transition state. In His-Cys homing endonucleases and *Serratia* nuclease, only one asparagine is involved in metal ion coordination. The coordinated metal ion is believed to stabilize the transition state by neutralizing the negatively charged pentavalent phosphoanion transition state. In ColE9, it has been proposed that the water molecule coordinated by the metal ion and His131 acts as the general acid that protonates the leaving group [29]. The conserved asparagine lowers the pKa of the invariable histidine which in turn activates the nucleophilic water molecule for in-line attack of the scissile phosphate. Recently, more Type II restriction endonucleases, namely, KpnI [36], MnlI [37], Hpy99I [35], Eco31I [38], [39], HphI [40], SphI [41], PacI and others [42] are identified as containing this HNH motif through X-ray crystallography or sequence alignment/structural prediction. GIY-YIG endonucleases (including homing endonucleases I-TevI [43], [44], nucleotide excise repair enzyme UvrC and Type IIP REases Hpy188I [45] Eco29kI [46], [47] and Cfr42I [47], [48]) is proposed to adapt a similar catalytic mechanisms as HNH/His-Cys endonuclease except for the use of Tyr as the general base based on the structure of UvrC [49].

In this study, we demonstrated the importance of conserved catalytic residues H294, H295, N311, and H320 by site-directed mutagenesis. HpyAV variants H294D, N311A, and H320A lack any detectable catalytic activity (less than 0.1% activity). Substitution of the general base His295 by Ala displays impaired cleavage activity only (H295A retains approximately 1% of wt activity), suggesting that an alternative weaker general base exists in the catalytic site when the general base His295 is mutated. In addition, positively charged aa substitution by Lys in H295K did not rescue the catalytic activity, indicating Lys cannot replace His as the general base in HpyAV. Other charged aa residue substitutions at the 295 position (HpyAV variants H295N, H295D, and H295E) failed to show any detectable cleavage activity. The observation that deletion of the proposed histidine general base in the HpyAV active site yields an endonuclease with reduced activity, rather than completely inactivating the enzyme, is unusual for the HNH superfamily, but not unprecedented. Mutation of the active histidine general base in the I-PpoI homing endonuclease also results in reduced activity [50]; this result is attributed to the ability of a neighboring histidine to participate in a less-efficient long-range proton transfer reaction and activation of the nucleophilic water with reduced activity as compared to the wild-type enzyme.

MnlI and I-PpoI are the only ββα-Me endonuclease whose metal ion preference has been systematically studied. In the presence of 1 mM M^++^, the order of MnlI activity was shown to be Mg^++^ > Ni^++^  =  Co^++^ > Mn^++^ > Ca^++^ > Zn^++^ [51]. I-PpoI activity follows the order of Mg^++^ > Mn^++^ > Ca^++^  =  Co^++^ > Ni^++^ > Zn^++^ (10 mM) [52]. Hpy99I is active in Mg^++^ and Mn^++^ but not in Ca^++^ or Zn^++^ [35]. It has also been reported that ColE9 prefers Mg^++^ and Ca^++^ for dsDNA and Ni^++^ for ssDNA substrates [18], [34], [53]. Our preliminary results showed that PacI and SphI are active with Ni^++^ but less so than in Mg^++^, whereas HphI showed comparable activity with Ni^++^ and with Mg^++^ (data not shown). Non-specific HNH endonucleases colicin E9 has also been reported to have distinct metal ion preference: Mg^++^ and Ca^++^ are most efficient cofactors for cleavage of double-strand DNA but Ni^++^ is most efficient for cleavage of single-strand DNA; and colicin E9 cleaves RNA in the absence of any divalent metal ions [34]. In addition, although *Serratia* endonuclease is most active with Mg++, mutants that are more active in Mn++, Co++ and Zn++ have been isolated [54]. Compared to our results reported here, where HpyAV activity follows the order of Mn^++^  =  Ni^++^  =  Co^++^ > Cu^++^ > Zn^++^ > Ca^++^ > Mg^++^ (2 mM for Mg^++^, Ca^++^, Mn^++^, Ni^++^ and Co^++^; 0.1 mM for Cu^++^ and Zn^++^) and KpnI activity follows the order of Mg^++^ > Co^++^ > Ni^++^  =  Zn^++^ > Ca^++^, it appears that HNH endonucleases in general have a less stringent metal ion requirement than their counterparts with the canonical PD-X-(D/E)XK catalytic motif.

HpyAV is unique in that it is the only HNH-type endonuclease characterized so far to have higher double-strand cleavage activity with transition metals (Mn^++^, Ni^++^ and Co^++^) than with Mg^++^. It has been argued that in ββα-Me REases, the metal ion is not involved in the coordination of the nucleophilic water but only interacts with the phosphoanion transition state and the leaving group through a water molecule, therefore allowing for a less stringent metal ion requirement for catalysis [55], [56]. This property also raises the caution that transition metal ions should be taken into consideration when the cleavage activity of HNH enzymes is to be optimized.

The loss of fidelity of KpnI in the presence of Mn^++^ has also been observed in EcoRI [57], PstI [58], EcoRV [59] and CeqI [60] and other DNA enzymes such as HIV ribonuclease H [61], the translesion DNA polymerase Dpo4 [62] and Tn10 in IS10 transposition [63]. This effect has been attributed to the similar chemical nature of Mn^++^ to Mg^++^ but a relaxed coordination requirement for Mn^++^ [55], suggesting a role of the metal ion for the specificity of these REases. In EcoRV, it has also been demonstrated that the affinity for Mg^++^ is lower when the enzyme is bound to non-cognate sites [64]. Interestingly, HpyAV does not show increased non-cognate cleavage activity in Mn^++^ under our assay conditions, possibly because unlike Type IIP REases, target site recognition and phosphodiester bond cleavage of Type IIS REases such as HpyAV are uncoupled by separate DNA recognition and cleavage domains. The different metal ion preference of different HNH endonuclease is an interesting phenomenon, given their highly similar set of metal coordinators and general base. Further genetic, biochemical and structural studies of HNH enzymes are needed to understand the catalytic role of different metal ions. For example, it is possible to target the metal binding region by localized saturation mutagenesis of HpyAV by construction of a plasmid mutant library in the presence of methylase protection and then transfer the mutant library DNA into non-modified *dinD::lacZ* indicator strain and screen for blue colonies on X-gal plates supplemented with high concentration of Mg^++^ (active mutants will damage chromosomal DNA and induce SOS-induction *in vivo*). Such active HpyAV mutants may contain altered metal binding site with preference for Mg^++^ as a cofactor. KpnI mutants with altered metal binding and preference have been isolated (SHC and SYX, unpublished results).

### Distribution of HpyAV Homologous Systems

In addition to Hin4II, a BLASTP search of GenBank database discovered 10 putative R-M systems highly homologous to the HpyAV system. Interestingly, these R-M systems are mostly carried by infectious microorganisms of human or mammalian hosts closely associated with humans (Table 2). While *Helicobacter pylori* strains where HpyAV and the putative HpyPORF28P and HpyGORF49P R-M systems reside, are the etiological agent of common gastritis and a risk factor for gastric cancer, *Campylobacter upsaliensis*, *Yersinia kristensenii* and *Vibrionales bacterium* are mainly zoonotic but opportunistic pathogens of humans. *Bacteroides stercoris* is a symbiotic bacterium in the human intestines that helps to digest food. *Haemophilus influenzae*, from which Hin4II is isolated, is found in the upper respiratory tract of humans; it can cause bacteremia, pneumonia and acute bacterial meningitis. *Neisseria meningitidis* causes meningitis in humans. Other HpyAV homologous systems are found in microbes that infect pigs (enteric *Brachyspira hyodysenteriae*) [65], cattle, goats (*Mycoplasma mycoides* through inhalation) [66], [67] and horses (*Streptococcus equi* that causes strangles) [68]. It is possible that these pathogenic microbes acquired a prototypical R-M system through lateral transfer when they were brought in the vicinity of a mammalian host organism, which need not be permissive to the microorganisms concerned [69], [70]; or they could be transferred during food intake. It has been shown that the virulent factor iceA1 is a functional isoschizomer of NlaIII in *H. pylori* CH4 [71], [72]. Therefore, the acquired HpyAV homologous systems may provide survival advantage to the receiving microorganisms. It is noted, however, that whole-genome sequencing efforts have largely been focused on mammalian pathogens and their sequences are over-represented in sequence databases. It is possible that HpyAV homologous R-M systems exist in non-mammalian-associated microorganisms. Shotgun sequencing of marine samples has revealed two ORFs EsaSS23P and EsaSS44P that are also homologous to HpyAV and Hin4II aa sequences.

## Materials and Methods

### Strains, DNA Sequences and Mutagenesis

The HpyAV R-M system was identified in *Helicobacter pylori* 26695 (Genbank nucleotide accession NC_000915). ORF hp_0054 is the M gene which was amplified in PCR and inserted into pSYX20 at the EcoRV and SphI sites with a GGAGGT ribosome-binding site and upstream stop codons in all three ORFs (pSYX20 carries pSC101 replication origin, Km^R^, and Tc^R^). Expression of the M gene is under the control of the Tc^R^ promoter. ORF hp_0053 is the R gene which was amplified in PCR and inserted into pAII17 (NEB) at NdeI and BamHI sites, under the control of the T7 promoter. The over-expression strain was constructed by sequential transformation of *E. coli* ER3081 (NEB) by pSYXS20-*hpyAVM* and then pAII17-*hpyAVR*. ER3081 (*fhuA2 8 lacZ::T7 gene1 [lon] ompT gal attB::*pCD13(*Ptet-lysY*, *lac*I^q^) [Spec^R^] *sulA11 R(mcr-73::miniTn10—*Tet^S^)*2 [dcm] R(zgb-210::Tn10 —*Tet^S^ *) endA1 *Δ*(mcrC-mrr)114::IS10)* is a derivative of ER2566 (T7 Express, NEB). This strain contains the T7 RNA polymerase gene at the chromosomal *lac* locus, replacing much of *lacZY*; the K128Y mutant of T7 lysozyme (*lysY*) and the *lacI^q^* gene are expressed from the chromosomal *attB* site. Stable integration of the *lysY* and *lacI^q^* genes was accomplished using the pCD13PKS plasmid described by Platt *et al*. [73], [74]. Site-directed mutagenesis was carried out using a modified inverse PCR procedure [75] using pAII17-*hpyAV*R isolated from the over-expression strain as template. Primers designed to construct mutants (H295A, H320A and N311A) were synthesized by Integrated DNA Technologies. All DNA sequences were verified by DNA sequencing.

### Protein Expression and Purification

The over-expression strain of HpyAV was cultured in LB medium containing 100 µg/ml ampicillin and 30 µg/ml kanamycin at 30°C and 200 rpm overnight (∼15 h). Ten milliliters of the overnight culture was inoculated into 1 L of LB medium containing the same antibiotics and cultured at 30°C and 200 rpm to log phase. The culture was cooled down to 25°C before IPTG was added to a final concentration of 0.25 mM. Growth was then continued at 25°C for ∼15 h and the cultures were harvested by centrifugation. The cell pellet was resuspended in 100 ml of 20 mM Tris-HCl, pH 8.0, 50 mM NaCl, 1 mM EDTA (Buffer A) supplemented with 1% PMSF and sonicated on ice. After centrifugation, the supernatant was loaded onto a Heparin HiTrap column (5 ml; GE Life Sciences). Peak fractions from a linear elution gradient of 0.05–1 M NaCl in Buffer A was diluted 4-fold in Buffer A and loaded onto a HiTrap SP HP column (5 ml; GE Life Sciences). Peak fractions from a 0.05–1 M NaCl gradient were pooled and concentrated by Vivaspin 15 (10 kDa MWCO; Sartorius). An equal volume of 60% glycerol was added to the concentrated protein for storage at −20°C.

### DNA Cleavage Activity Assays

The DNA cleavage activity of the crude extract or purified HpyAV was assayed in 50 µl reactions containing 20 mM Tris-HCl, pH 7.9, 200 mM NaCl supplemented with the indicated concentrations of MgSO_4_, CaCl_2_, MnCl_2_, CoSO_4_, NiSO_4_, Cu(OAc)_2_ or Zn(OAc)_2_ and 1 µg of λ DNA at 37°C for 1 h. KpnI was assayed in 20 mM Tris-HCl, 50 mM NaCl, pH 7.9 with the same battery of salts using 1 µg of pXba DNA (a 10 kb XbaI fragment of adenovirus DNA inserted into pUC19; NEB). EcoRI activity assay was carried out in the same buffer using 1 µg of λ DNA. The cleavage reactions were then analyzed by 1.2% agarose gel electrophoresis in 1x TBE. One enzyme unit is defined as the amount of enzyme needed to cleave the 1 µg of the designated DNA completely at 37°C in 1 h. Specific activity is defined as the number of units per mg of enzyme. Specific activity was determined in duplicate (KpnI) or triplicate (HpyAV) by titrating the enzymes (in steps of 2-fold dilution) in their respective reaction buffer in the presence of 2 mM MgSO_4_, CaCl_2_, MnCl_2_, CoSO_4_ or NiSO_4_, or 0.1 mM Cu(OAc)_2_ or Zn(OAc)_2_. KpnI and EcoRI were from NEB. All reaction buffers and metal ion solutions were prepared using MilliQ water.

### Homology Modeling and Structural Alignment

Amino acid residues 281 to 360 of HpyAV were modeled to the ββα-Me motif of ColE9 (mutant H103A; PDB: 1V14) by homology modeling using SWISS-MODEL [76], [77]. The structural model of the HpyAV ββα-Me motif was aligned pair-wise with the crystal structures of ColE9 (PDB: 1V14), I-HmuI (PDB: 1U3E), Hpy99I (PDB: 3GOX) and the KpnI model built by Nagaraja and colleagues [36] using the TM-Align module [78] of STRAP [79].
